# Increased dose near the skin due to electromagnetic surface beacon transponder

**DOI:** 10.1120/jacmp.v16i3.4930

**Published:** 2015-05-08

**Authors:** Kang‐Hyun Ahn, Ryan Manger, Howard J. Halpern, Bulent Aydogan

**Affiliations:** ^1^ Department of Radiation Oncology St. Mary's Good Samaritan Inc. Centralia IL; ^2^ Department of Radiation Medicine and Applied Sciences University of California San Diego La Jolla CA; ^3^ Department of Radiation Oncology University of Illinois at Chicago Chicago IL; ^4^ Department of Radiation and Cellular Oncology University of Chicago Chicago IL USA

**Keywords:** Calypso, surface beacon, electromagnetic transponder, target localization, skin dose, Monte Carlo

## Abstract

The purpose of this study was to evaluate the increased dose near the skin from an electromagnetic surface beacon transponder, which is used for localization and tracking organ motion. The bolus effect due to the copper coil surface beacon was evaluated with radiographic film measurements and Monte Carlo simulations. Various beam incidence angles were evaluated for both 6 MV and 18 MV experimentally. We performed simulations using a general‐purpose Monte Carlo code MCNPX (Monte Carlo N‐Particle) to supplement the experimental data. We modeled the surface beacon geometry using the actual mass of the glass vial and copper coil placed in its L‐shaped polyethylene terephthalate tubing casing. Film dosimetry measured factors of 2.2 and 3.0 enhancement in the surface dose for normally incident 6 MV and 18 MV beams, respectively. Although surface dose further increased with incidence angle, the relative contribution from the bolus effect was reduced at the oblique incidence. The enhancement factors were 1.5 and 1.8 for 6 MV and 18 MV, respectively, at an incidence angle of 60°. Monte Carlo simulation confirmed the experimental results and indicated that the epidermal skin dose can reach approximately 50% of the dose at $d_{\text{max}}$ at normal incidence. The overall effect could be acceptable considering the skin dose enhancement is confined to a small area ($∼1\;{\text{cm}}^{2}$), and can be further reduced by using an opposite beam technique. Further clinical studies are justified in order to study the dosimetric benefit versus possible cosmetic effects of the surface beacon. One such clinical situation would be intact breast radiation therapy, especially large‐breasted women.

PACS number: 87.53

## I. INTRODUCTION

Real‐time motion tracking provides crucial clinical advantages for external‐beam radiation therapy by allowing clinicians to pursue more advanced treatment objectives with greater confidence. The Calypso 4D localization system (Calypso, Varian Medical Systems, Palo Alto, CA) was demonstrated to have potential to increase therapeutic ratio in prostate, pancreas, and lung treatments.^1^, ^2^, ^3^, ^4^, ^5^ Recent developments allow the use of a surface beacon transponder anywhere on the body, and the system has expanded motion monitoring and management techniques using noninvasive methods. One of the immediate applications of this versatile approach would be radiation therapy of breast cancer, the most common cancer in women. As this is commonly treated in supine position, respiratory motion in the chest and upper abdomen reaches up to 1.5 cm. Moreover, the breast, particularly a large breast, is a semisolid organ susceptible to large contour variations with small setup and respiratory variations. Real‐time motion tracking and appropriate beam hold‐off protocol may greatly improve the treatment reproducibility and, consequently, the accuracy of treatment delivery.^6^, ^7^ This is particularly important for treatments using sophisticated techniques, such as breath hold or hypofractionated approach.

Implanted fiducial markers with a high atomic number result in hot and cold spots at the upstream and downstream regions, respectively. Although these effects do not cancel out for an opposite beam pair and arc geometries, the overall clinical impact is generally considered insignificant.^8^, ^9^ Use of the electromagnetic surface beacon, however, potentially involves direct irradiation of the copper coil transponder placed on the patient's skin, which may incur unwanted skin reaction due to bolus effect of the transponder. To our knowledge, there is no published work that documents the surface dose enhancement of Calypso beacon. In this work, we evaluated the increase of skin dose due to the Calypso surface beacon transponder under various beam incidences and energies, using film measurements and Monte Carlo simulations.

## II. MATERIALS AND METHODS

**Figure 1:**
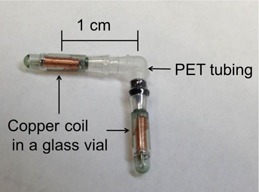
Calypso surface beacon transponder. Two transponders are assembled by a polyethylene terephthalate (PET) tubing.

Figure 1 shows the surface beacon device consisting of two transponders fabricated in an “L” shaped assembly. The two transponders have their spatial centers 1 cm from the center of the elbow, and resonate at unique frequencies. Each transponder measures 1.85 mm in diameter and between 8.0 and 8.7 mm in length. A black orientation indicator is visible on the low‐frequency arm to facilitate consistent orientation of the device on the patient's skin. Surface dose measurements were performed with a Varian 2100EX Clinac linear accelerator (Varian Medical Systems, Palo Alto, CA). The linac was calibrated to produce 0.85 cGy/MU for 6 MV and 0.9 cGy/MU for 18 MV at 10 cm depth, 90 cm source‐to‐surface distance (SSD), with 10 cm×10 cm field size. The surface beacon was placed in the center of the 10×10 cm field on a 30 cm×30 cm×8 cm solid water phantom at 100 cm SSD.

Kodak EDR2 film (Carestream Health, Rochester, NY) was placed between the surface beacon and the solid water phantom. The gantry was rotated to produce beam incidence angles of 0°, 20°, 40°, 60°, 80°, and 180° (backscatter) for both 6 MV and 18 MV. Each irradiation delivered 200 monitor units (MU), which would result in 216 cGy and 190 cGy at depth on maximum dose ($d_{\text{max}}$) at normal incidence for 6 MV and 18 MV, respectively. For film analysis, a calibration film from the same batch was irradiated at 10 cm depth in solid water with separate 5×5 cm fields of 50, 100, 150, 200, 250, and 300 cGy. More details of our film calibration is provided in a previous study.^(10)^ Field sizes and depths have little effect (<1%) on the sensitometric curves of EDR2 film.^(11)^ The films were developed with a Kodak X‐OMAT 3000RA processor, and scanned with an Epson Expression 10000XL photo scanner (Epson Singapore Pte., Ltd., Singapore, Singapore). The calibration curve was confirmed to measure dose within 2% accuracy up to a dose of 300 cGy.

A general‐purpose Monte Carlo code MCNPX (Monte Carlo N‐Particle) and Mohan 6 MV spectrum^(12)^ was used to perform simulations to supplement the experimental data. The accuracy of the simulation was confirmed by checking that the simulated percent depth dose (PDD) fell within 2% of that generated by our treatment planning system (Eclipse AAA, Varian Medical Systems, Palo Alto, CA) at depths greater than 5 mm. The errors were up to 6% for depths less than 5 mm. We modeled the surface beacon geometry using the actual mass of glass vial (20 mg) and copper coil (36 mg)^(13)^ placed in the L‐shape polyethylene terephthalate tubing. The simulation was carried out at depths of 0.2, 0.4, and 0.6 mm to match with the effective depth of surface dose measurement for film dosimetry. The physical thicknesses of the film and the envelope including the film were 0.19 mm and 0.69 mm measured by digital calipers, respectively, indicating its effective depth of measurement to be 0.35 mm. The dimension of energy deposition mesh tally was 1 mm×1 mm in plane, 0.2 mm thick in depth. The cutoff energy was 10 keV for both electron and photon. Number of histories was 8 billion, and the coefficients of variation ranged from 2.8% to 4.6%, depending on the voxel.

## III. RESULTS

**Figure 2:**
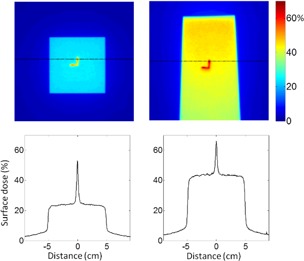
Shown are the surface dose measured by EDR2 film for 6 MV beam with incidence angle 0° (left) and 60° (right), on the top row. Below are the dose profiles for the plane shown with dotted lines on the dose profiles. Dose values are shown as percentage of the dose at the depth of 1.5 cm with normal incidence.

Film dosimetry revealed an increased surface dose under the Calypso beacon. In Fig. 2, the upper panels display 2D surface dose measurements with beam incidence angles at 0° (left) and 60° (right) for 6 MV. Dose values are shown as percentage of the dose at $d_{\text{max}}$ with normal incidence. Shown are the 1D dose profiles corresponding to the horizontal lines in the 2D dose maps. At normal incidence, the intrinsic surface dose away from the beacon was 24% of the dose at $d_{\text{max}}$, and had a steep increase below the beacon with a peak of 53% and 2.8 mm full width at half maximum (FWHM). At a 60° angle of incidence, the intrinsic surface dose and the beacon‐enhanced peak surface dose increased to 43% and 66%, respectively. The corresponding factors of dose enhancement ratio were 2.2 and 1.5 for the incidence angles of 0° and 60°, respectively.

**Figure 3:**
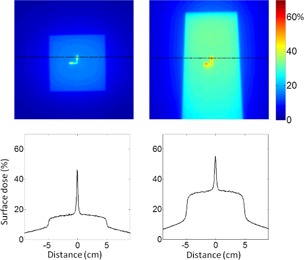
Surface dose measured by EDR2 film for 18 MV beam with incidence angle 0° (left) and 60° (right) on the top row. Below are the dose profiles for the plane shown with dotted lines on the dose profiles. Dose values are shown as percentage of the dose at the depth of 3 cm with normal incidence.

**Figure 4:**
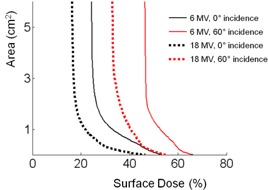
Dose‐area histogram for the dose distributions measured with films shown in 2, 3. Dose values are shown as percentage of the dose at $d_{\text{max}}$ with normal incidence.

Figure 3 shows the dose distributions for 18 MV beam within the same setting as Fig. 2. At the incidence angle of 0° and 60°, the intrinsic surface dose values were 16% and 32% of the dose at $d_{\text{max}}$, which increased to 46% and 55% below the beacon, respectively, with 2.8 mm FWHM. The corresponding factors of dose enhancement ratio were 3.0 and 1.8 for the incidence angles of 0° and 60°, respectively. Figure 4 shows a dose‐area histogram for the dose distributions shown in 2, 3. Most of the areas were covered by the intrinsic dose value, but a small, approximately 1 cm^2^ area, was subject to the bolus effect from the beacon. Compared to the normal incidence, the beams that are incident at 60° resulted in dose enhancement to a slightly wider area ($∼1.5\;{\text{cm}}^{2}$).

**Figure 5:**
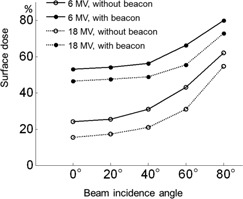
Variation of the surface dose with the beam incidence angle. Dose values were normalized to the dose at $d_{\text{max}}$ with normal incidence. The surface dose with and without the Calypso beacon are shown as open and closed circles, respectively.

Figure 5 shows the bolus effect of the surface beacon at various beam incidence angles for 6 MV and 18 MV X‐rays. The higher energy beam, shown with dotted lines, had greater relative enhancement from the bolus effect, whereas its surface dose values normalized to the dose at $d_{\text{max}}$ at the normal incidence were lower than those of 6 MV beam. Gantry rotation of 180° from the normal incidence setup measured beacon backscatter factors of 1.1 for the beams of either energy. The intrinsic surface dose in the absence of the beacon increased with the incidence angle. The dose below the Calypso surface beacon increased accordingly and reached up to ∼80% of the dose at $d_{\text{max}}$ at 80° angle. The dose enhancement, however, decreased gradually with the incidence angle. While maximum dose enhancements of 2.2 for 6 MV and 3.0 for 18 MV were observed at normal incidence, both were reduced down to 1.3 at 80° angle.

**Figure 6:**
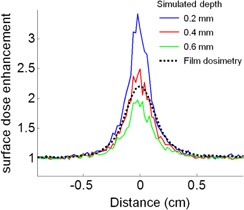
Surface dose enhancement determined with film and Monte Carlo simulation for the 1D surface dose profile shown in Fig. 2 (normal incidence, 6 MV). The dose enhancements from the surface beacon were obtained at effective depths of 0.2 (blue), 0.4 (red), and 0.6 mm (green) by MC simulation. The dose measured with film is shown with a dotted line.

The 1D surface dose profile for a normally incident 6 MV beam was further examined using Monte Carlo simulation at various effective depths of measurement. Each simulated dose profile in Fig. 6 represents enhancement in the dose deposited within 0.2 mm thick layers, where the depth of the layer, defined as the distance between the layer's center and the surface, was 0.2, 0.4, and 0.6 mm. The simulated dose enhancement under the surface beacon was 3.4 at 0.2 mm depth, and gradually decreased to 2.4 and 1.9 at depths of 0.4 mm and 0.6 mm, respectively. Film‐measured dose profile had a comparable full width at half maximum and peak dose value to the MC simulated dose deposition at 0.4 mm depth, which is the effective depth of film measurements.

**Figure 7:**
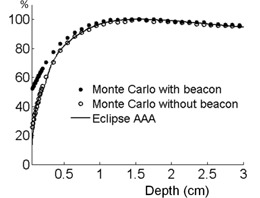
PDD for 6 MV beam simulated with Monte Carlo simulation and AAA algorithm (Varian Eclipse Medical Systems, Palo Alta, CA).

Figure 7 shows a PDD comparison for a normally incident 6 MV beam. Closed and open circles represent the Monte Carlo simulation with and without the surface beacon, respectively. The PDD generated by our treatment planning system is shown as a solid line and is well compared to the Monte Carlo simulation without the beacon. Measured PDD is not shown as it is not distinguished from the treatment planning system's PDD in this scale. The simulated PDDs indicate substantial bolus effect over the depth less than 5 mm. At depths deeper than 5 mm, the surface beacon did not have noticeable effect on the PDD.

## IV. DISCUSSION & CONCLUSIONS

The outer layer of the human skin, epidermis, consists of 10 to 20 layers of keratinizing cells with the actively proliferating compartment located at 0.03–0.3 mm depth.^(14)^ In particular, for the breast, the epidermis thickness is about 0.045 mm.^(15)^ This site of early radiation reactions is the major beneficiary of the skin‐sparing properties of megavoltage beams. Use of high atomic number (Z) fiducials, such as the Calypso surface beacon, however, may compromise the skin sparing at the depths relevant to the early response. In this regard, our study of film dosimetry and simulation provides useful dosimetric data under different beam geometries and energies.

The Calypso surface beacon consists of two 36 mg copper coils for wireless electromagnetic localization. Upon irradiation with megavoltage photon beams, these relatively high‐Z materials attenuate the primary beams and, concurrently, produce more secondary electrons to increase local deposition of the dose.^8^, ^9^ Surface dose in the buildup regions is dominated by the latter (bolus effect) and, accordingly, we observed a substantial increase in surface dose with film measurement, which was verified with the Monte Carlo simulation.

Figure 5 shows that the relative dose enhancement was more conspicuous with the higher photon energy. This is due to increasing component of pair‐production, and is consistent with a previous interface dosimetry study with high‐Z material.^(16)^ However, due to the stronger skin‐sparing effect of higher energy, surface doses with 18 MV X‐rays were actually less than that of 6 MV with the values normalized to the dose at $D_{\text{max}}$. Nevertheless, the beacon substantially spoiled the skin‐sparing effect of high‐energy photons. The normal incidence surface dose of 18 MV, 46% of the dose at $d_{\text{max}}$, due to the beacon, was much greater than the generic beam sparing effect of 6 MV, 24% of the dose at $d_{\text{max}}$, without the beacon. On the other hand, with the increasing incidence angle, the intrinsic surface dose went up as more electrons are set in motion along the oblique path of the beam at shallower depth.^(17)^ EDR2 film is known to have an intrinsic directional sensitivity, and the dose can be underestimated by 4% with parallel incidence.^(18)^ Although this was not taken into account in our analysis, the overall trend indicates that the contribution from the surface beacon was relatively reduced at high‐incidence angle.

It should be noted that the effective point of measurement of 0.35 mm for the EDR2 film may be slightly deeper than that of the epidermis basal layer. Correspondingly, the simulated 1D profile at 0.4 mm depth compares well to the film dosimetry, as shown in Fig. 6. The epidermal skin dose at 0.1 mm can be inferred from the Monte Carlo study. The simulated PDDs in Fig. 7 indicate it can reach approximately 50% of the dose at $D_{\text{max}}$.

Use of a surface beacon for motion monitoring may well predispose the transponder to be positioned within the treatment field. Although our results indicate factors of 2∼3 increase in the surface dose due to the bolus effect of the beacon, the enhancement is confined to a small area (Fig. 4) and the relative effect would be further reduced with the use of an opposite beam‐pair technique. For example, consider a hypothetical opposite beam‐pair situation for a breast treatment where the beacon is placed within the field and is irradiated by a beam with 40° incidence angle. The beacon could enhance the surface dose by a factor of 1.8 (Fig. 5) for the incident beam, and by 1.1 for the exit beam backscattering. Any undesirable dose enhancement may be minimized by moving the beacon to a different position for each fraction. In practice, even without the active attempts to move, there would be some feathering effects that mitigate the dose enhancement due to random errors associated with the beacon placement for each fraction. Furthermore, it is usually possible to identify an appropriate site of placement for the surface beacon to avoid direct irradiation. The Calypso system requires that the transponder is within a $14\times 14\;{\text{cm}}^{2}$ localization volume in the lateral and longitudinal directions with respect to the center of the flat‐panel electromagnetic array, which, in turn, is aligned to the treatment isocenter laterally and longitudinally. If the field size is smaller than the functional region of the electromagnetic array, the surface beacon can be placed outside the treatment field and, at the same time, can achieve pertinent monitoring of the organ or tissue motion by exploiting the size of the functional volume of the array. If it is not possible to move the beacon outside of the treatment field, it may be moved away from the cosmetically more sensitive upper inner quadrant of the breast.

There may be a valid dose homogeneity benefit and reduction of dose to critical structures from use of the Calypso system. With better definition of the radiation dose distribution, hot spots may be avoided, as regions of low separation enter into regions more heavily blocked in the treatment plan. Large breasts present the situation of a large tissue mass often relatively weakly coupled to the thoracic core. This can produce substantial tissue shifts during respiration, or with small patient motions during treatment. Dynamic monitoring with Calypso may avoid the dosimetric consequences of such shifts, which would otherwise have resulted in hot spots in the low separation regions. This will enhance accuracy of the treatment plan delivery, which is optimized to minimize dose heterogeneity and to avoid excessive exposure of critical tissue. Historic data indicate a strong dependence of skin sequellae on both dose and exposed surface areas.^(14)^ Although the skin sequellae may be small considering the size of the beacon's bolus effect, avoiding unplanned “hot spots” may provide enhanced cosmetic benefit.

A clinical study designed to determine acute skin reactions due to the use of the Calypso beacon would provide instrumental information concerning its dosimetric consequences. If minimal skin reaction is observed, then the benefits can be pursued with confidence. Even if there are some increased skin reactions, depending on their severity, a study to evaluate the long term risk of skin sequellae versus benefit of using the Calypso surface beacon for organ motion management should be considered.
